# Evolutionary Regression and Species-Specific Codon Usage of TLR15

**DOI:** 10.3389/fimmu.2018.02626

**Published:** 2018-11-13

**Authors:** Carlos G. P. Voogdt, Mark E. Merchant, Jaap A. Wagenaar, Jos P. M. van Putten

**Affiliations:** ^1^Department of Infectious Diseases and Immunology, Utrecht University, Utrecht, Netherlands; ^2^Department of Chemistry, McNeese State University, Lake Charles, LA, United States; ^3^Wageningen Bioveterinary Research, Lelystad, Netherlands

**Keywords:** toll-like receptor, TLR15, reptile, codon-bias, protease activated receptor

## Abstract

Toll-like receptors (TLRs) form an ancient family of innate immune receptors that detect microbial structures and activate the host immune response. Most subfamilies of TLRs (including TLR3, TLR5, and TLR7) are highly conserved among vertebrate species. In contrast, TLR15, a member of the TLR1 subfamily, appears to be unique to birds and reptiles. We investigated the functional evolution of TLR15. Phylogenetic and synteny analyses revealed putative TLR15 orthologs in bird species, several reptilian species and also in a shark species, pointing to an unprecedented date of origin of TLR15 as well as large scale reciprocal loss of this TLR in most other vertebrates. Cloning and functional analysis of TLR15 of the green anole lizard (*Anolis carolinensis*), salt water crocodile (*Crocodylus porosus*), American alligator (*Alligator mississippiensis*), and chicken (*Gallus gallus*) showed for all species TLR15 specific protease-induced activation of NF-κB, despite highly variable TLR15 protein expression levels. The variable TLR15 expression was consistent in both human and reptilian cells and could be attributed to species-specific differences in TLR15 codon usage. The species-specific codon bias was not or barely noted for more evolutionarily conserved TLRs (e.g., TLR3). Overall, our results indicate that TLR15 originates before the divergence of chondrichthyes fish and tetrapods and that TLR15 of both avian and reptilian species has a conserved function as protease activated receptor. The species-specific codon usage and large scale loss of TLR15 in most vertebrates suggest evolutionary regression of this ancient TLR.

## Introduction

Toll-like receptors (TLRs) are innate immune receptors that have a critical role in the early detection of infection (1). The general architecture of TLRs consists of a ligand-binding extracellular domain containing multiple leucine rich repeats (LRR), a single transmembrane domain and an intracellular Toll-interleukin-1 (TIR) signaling domain (2). Ligand-induced TLR signaling activates immune-related transcription factors (e.g., nuclear factor κB, NF-κB) which induce expression of pro-inflammatory genes. The importance of TLRs in the immune system is underlined by the strong evolutionary conservation of this family of receptors. The prototypical Toll receptor originates at the base of metazoa ~600 million years ago (3). Subsequently, extensive gene duplication and gene loss events have resulted in 10 different TLRs in some mammals (including humans) to more than 20 TLRs in teleost fish (4–6). Evolutionary diversification of the TLR ligand-binding domain to detect diverse types of microbial structures has resulted in distinct TLR subfamilies.

One of the TLR subfamilies that has evolved highly dynamically is the TLR1 subfamily that comprises TLR1, TLR2, TLR6, and TLR10. Members of this subfamily typically function as heterodimeric receptors. Heterodimers of TLR2 and TLR1 or TLR6 respond to microbial lipopeptides such as Pam_3_CSK_4_ (TLR2/TLR1) or FSL-1 (TLR2/TLR6) (7–9). In mammals TLR1 and TLR6 arose by tandem duplication and are limited in divergence due to gene conversion (10). For reasons unknown, TLR10 has been preserved in some mammals (including humans) and has been lost in other vertebrates. Among teleost fish, the common carp (*Cyprinus carpio*) duplicated its TLR2 gene (11), whereas the Atlantic cod (*Gadus morhua*) lost TLR1, TLR2, TLR6, and TLR10 altogether (12). In birds, duplications of TLR1 and TLR2 are abundant (6, 13) which has left chicken (*Gallus gallus*) with the paralogs TLR2A, TLR2B, TLR1A (also known as TLR16), and TLR1B. Chicken TLR2B/TLR1A heterodimers show dual recognition of the Pam_3_CSK_4_ and FSL-1 ligands (14). Interestingly, one gene duplicate within the TLR1 subfamily that appears to have evolved independently of other TLR1 subfamily members is TLR15. This TLR functions as a homodimer rather than as a heterodimer and signals upon proteolytic cleavage of its extracellular domain by microbial proteases (15, 16). TLR15 is absent in mammals and was first described in chicken (17). A partial related sequence has also been identified in the genome of the reptile *Anolis carolinensis* suggesting that TLR15 may be unique to the reptilian lineage (16).

Reptiles can be broadly subdivided in lepidosauria (lizards, snakes, amphisbaenians, and tuatara) and archosauria (crocodiles and birds). The position of turtles among reptiles is still debated but molecular analyses tend to group turtles within archosauria (18). Reptiles were the first vertebrates that could permanently colonize terrestrial habitats and thereby came into contact with prehistoric terrestrial microbiota which shaped the immune system of reptiles and descending animals. Despite its central role in vertebrate evolution little is known about the reptilian immune system [but see (19–22)], especially at the level of reptile-microbe interactions. Previously, we unveiled adaptive evolution of TLR5 of the *A. carolinensis* lizard indicating different sensitivity of lizard and human TLR5 to bacterial flagellins (23). Given the dynamic evolution of TLR1 subfamily members and the recent increase in available whole genome sequences of reptiles and other non-mammals, we here aimed to define the extent of genomic and functional evolutionary conservation of TLR15 in non-avian reptiles.

Bioinformatics analyses uncovered the presence of TLR15 outside the reptilian lineage, as well as loss of TLR15 within the reptilian lineage. Functional activation assays with recombinant lepidosaurian (*A. carolinensis*) and archosaurian (*Crocodylus porosus* and *Alligator mississippiensis*) TLR15 revealed conservation of function among reptilian and chicken TLR15 orthologs. Markedly variable expression efficiency of different reptilian TLR15s in both human and reptilian cells could be experimentally attributed to species-specific codon usage of the respective TLR15 genes. Finally, interspecies variability of codon usage in TLR15 was higher compared to TLRs which evolved more stably within vertebrates.

## Results

### Identification of TLR15 in vertebrates

**Figure 1:**
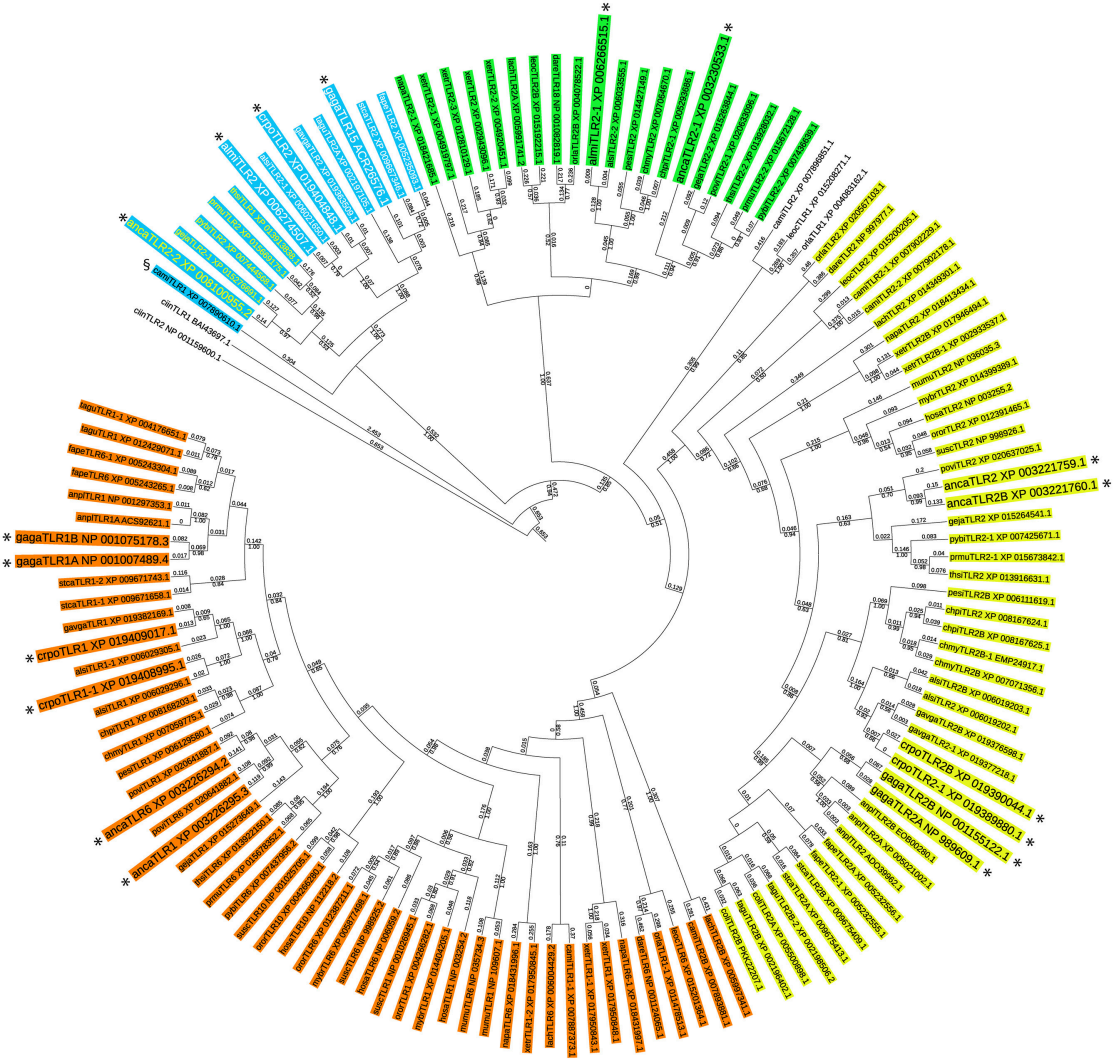
Phylogenetic tree of the vertebrate TLR1 subfamily. The evolutionary history of the vertebrate TLR1 subfamily was inferred by using the Maximum Likelihood method based on the JTT matrix-based model (24). The tree with the highest log likelihood (−28950.33) is shown. For each TLR sequence the GenBank accession number is indicated. For species abbreviations see Supplementary Table 1. Bootstrap analysis was performed with 250 iterations and the fraction of trees in which the associated taxa clustered together is shown below the branches (only bootstrap values greater than 0.5 are shown). Branch lengths indicate the number of substitutions per site and are shown above the branches. The analysis involved 136 full length amino acid sequences. All positions containing gaps and missing data were eliminated. There were a total of 252 positions in the final dataset. Evolutionary analyses were conducted in MEGA7 (25). The tree was customized using iTol (26). Four separate groups within the TLR1 subfamily are identified; (i) TLR2 (yellow), (ii) TLR1/6/10 (orange), (iii) a group of TLR2 present only in fish, amphibians, and non-avian reptiles (green), and (iv) the group containing chicken TLR15 (blue). In the TLR15 branch, lepidosaurians are show in yellow letters and archosaurians are shown in white letters. The predicted TLR1 of the ghost shark (*Callorhinchus milii*, camiTLR1) is indicated with the § symbol. All TLR1 subfamily members of *Anolis carolinensis* (anca), *Crocodylus porosus* (crpo), *Alligator mississippiensis* (almi), and *Gallus gallus* (gaga) are shown enlarged and are indicated with an asterisk.

In order to identify potential TLR15 sequences in vertebrate genomes we investigated the evolutionary relationship among TLR1 subfamily members from a diverse set of species (Supplementary Table 1) using a maximum likelihood based phylogenetic tree (Figure 1). Predicted TLR1 and TLR2 sequences of the uro-chordate *Ciona intestinalis*, an invertebrate, were used to root the tree. Analysis of the tree revealed four separate branches within the TLR1 subfamily; (i) TLR2, (ii) TLR1/6/10, (iii) a second group of TLR2 present only in fish, amphibians, and non-avian reptiles, and (iv) a group containing chicken TLR15 (Figure 1). An additional sequence more distantly related to the TLR2 precursor was found in the Australian ghost shark, spotted gar, and medaka but not in other vertebrates. Due to low sequence homology, the TLR15 branch did not contain TLR sequences of teleost fish, coelacanth, amphibians, or mammals. On the contrary, several sequences of birds as well as reptiles annotated in the database as TLR1 or TLR2 showed highest homology to chicken TLR15 and thus grouped in the TLR15 phylogenetic branch. This supports the notion that TLR15 is unique to the reptilian lineage. Unexpectedly however, a predicted TLR1 sequence of the Australian ghost shark (*Callorhinchus milii*) also clustered with high bootstrap support within the chicken TLR15 branch. Apart from the shark sequence, the TLR15 phylogeny recapitulates with high support the division of reptilia into lepidosauria and archosauria (Figure 1). Yet, annotated TLR1, 2 or 6 sequences of the bearded dragon (*Pogona vitticeps*, a lizard) and three species of turtles could be placed somewhere in the tree but none of the TLR sequences of these reptiles clustered within the TLR15-containing branch. This suggests that TLR15 has been lost from most non-reptilian lineages as well as from specific reptilian lineages after the divergence of lepido- and archosaurians.

**Figure 2:**
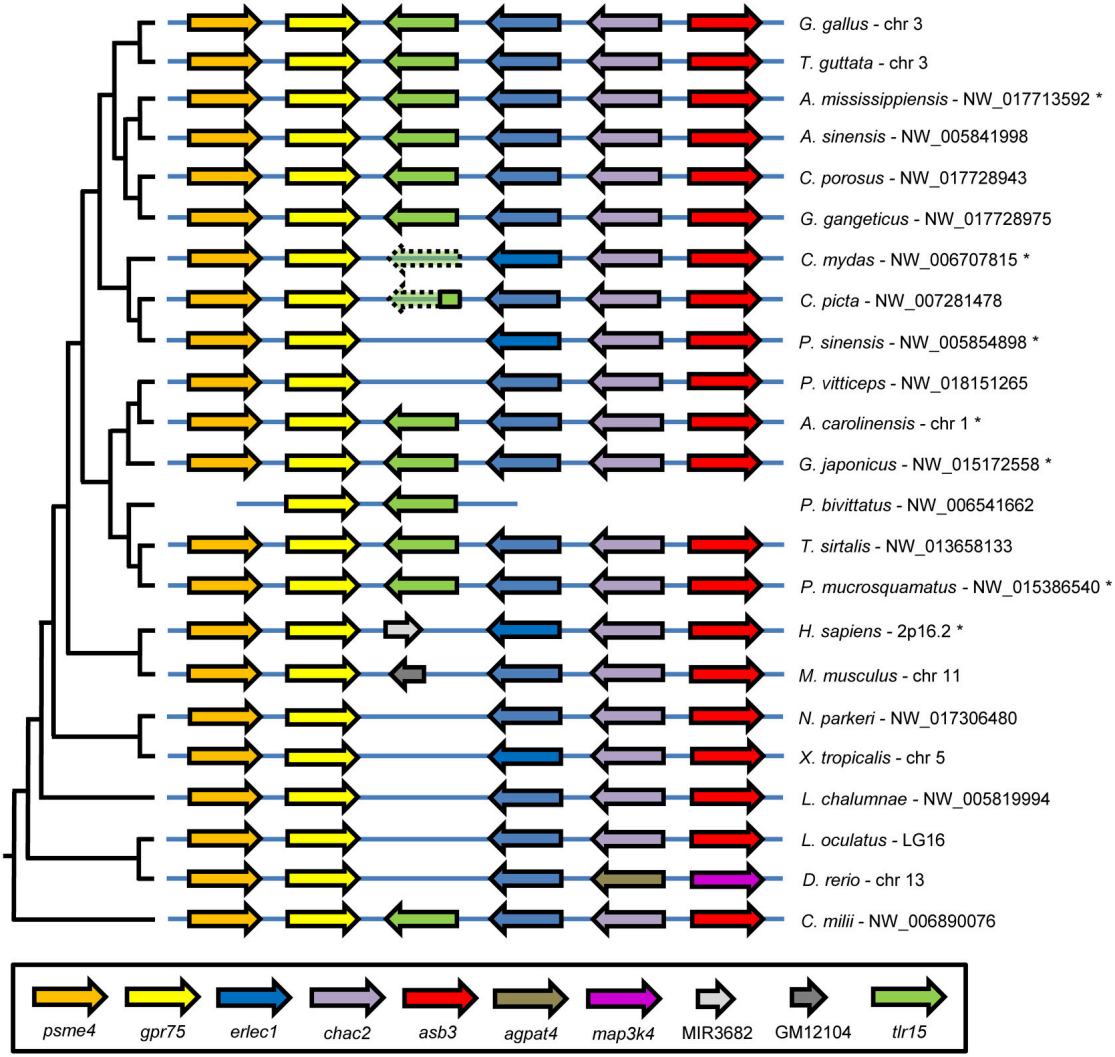
TLR15 gene synteny comparison among vertebrates. The genomic region containing chicken (*G. gallus*) *tlr15* compared to the same genomic region of the indicated species shows very high conservation of gene synteny. Genomic regions were collected from the NCBI Gene database and ordered according to the species phylogeny, shown on the left. Following the scientific name of each species is the NCBI Gene database identifier for this genomic region. *Tlr15* is shown in green and putative *tlr15* pseudogenes in the genomes of the *C. mydas* and *C. picta* turtles are shown in transparent color with a dashed line. Genes in this conserved genomic region are: proteasome activator complex subunit 4 (psme4), G-protein coupled receptor 75 (gpr75), endoplasmic reticulum lectin 1 (erlec1), ChaC cation transport regulator homolog 2 (chac2), ankyrin repeat and SOCS box containing 3 (asb3), 1-acylglycerol-3-phosphate O-acyltransferase 4 (agpat4), mitogen-activated protein kinase kinase kinase 4 (map3k4), microRNA 3682 (MIR3682). GM12104 in the mouse genome is a pseudogene without TLR features. Genomic regions of species indicated with an asterisk are annotated in NCBI's Gene bank database in reverse order.

To gain additional evidence for reciprocal loss of TLR15 in teleost fish, amphibians, mammals, turtles, and the bearded dragon and to confirm the conservation of putative TLR15 in the other reptiles, we collected the genomic region surrounding *tlr15* from the NCBI Gene database and compared the gene synteny in this region between chicken and other species. This showed that chicken *tlr15* is flanked by *psme4, erlec1, gpr75, chac2*, and *asb3*. These genes are absolutely conserved and arranged in this order in all species investigated here, except for zebrafish in which *chac2* and *asb3* are replaced by *agpat4* and *map3k4* (Figure 2). While all five genes surrounding chicken *tlr15* are conserved in the same order in the bearded dragon, no gene was identified between *erlec1* and *gpr75* in this reptilian species. The same was true for the Chinese softshell turtle. In the green sea turtle a predicted TLR2 pseudogene with high homology to chicken TLR15 is situated between *erlec1* and *gpr75*. In the genome of the painted turtle, a 214 amino acid coding sequence is conserved between *erlec1* and *gpr75* that has high homology to the TIR domain of chicken TLR15 and thus may represent a remnant of TLR15. Teleost fish, coelacanth, and amphibians carry no genes between *erlec1* and *gpr75*. In humans a microRNA encoding sequence is present at this position and in mice a pseudogene is predicted at this location but the residual protein sequence lacks leucine rich repeats, transmembrane regions or a TIR domain. Conversely, all of the predicted TLR1 or TLR2 sequences of species that grouped in the TLR15 branch of the phylogenetic tree, including the predicted TLR1 of the ghost shark (Figure 1), mapped between *erlec1* and *gpr75* (Figure 2).

Together, the phylogenetic and synteny analyses strongly suggest that the precursor of TLR15 is an ancient gene duplicate of the TLR2/1/6/10 precursor dating back at least to the common ancestor of chondrichthyes fish and tetrapods and that TLR15 has been reciprocally lost from the teleost fish, coelacanth, amphibian, mammalian, and even specific reptilian lineages.

### Cloning and characteristics of reptilian TLR15

Gene evolution is largely driven by selection on function. We therefore investigated whether the putative TLR15 genes found in lepidosaurian and archosaurian reptiles still encode a functional receptor. Hereto, putative *tlr15* genes were amplified from DNA of the lepidosaurian *Anolis carolinensis* (ancaTLR15) and archosaurians *Crocodylus porosus* and *Alligator mississippiensis* (crpoTLR15 and almiTLR15 resp.). Genes were cloned upstream of a C-terminal hemagglutinin (HA) or FLAG-tag sequence in an expression vector. Reptilian *tlr15* genes comprise a single exon and encode proteins of 823 (ancaTLR15), 877 (crpoTLR15), and 875 (almiTLR15) amino acids in length. Comparison of the putative TLR15 protein sequences of reptiles and chicken (gagaTLR15, 868 amino acids) showed that both full length crocodilian TLR15 proteins are more similar to chicken (69%) than to anolis TLR15 (59%) (Supplementary Table 2). All proteins had a similar architecture consisting of an extracellular domain (ECD) with 20 (gagaTLR15, crpoTLR15, and almiTLR15) or 18 (ancaTLR15) leucine rich repeats (LRRs), a C-terminal LRR (CTLRR), a single transmembrane region and a highly conserved intracellular TIR domain. Like TLR1, TLR6, and TLR10 of other species, all TLR15 sequences lack a cysteine containing N-terminal LRR (NTLRR) and like TLR2, the CTLRR of TLR15 is characterized by a CxCx24Cx20C cysteine motif (27) (Supplementary Figure 1).

### Activation of reptilian TLR15 by proteases

**Figure 3:**
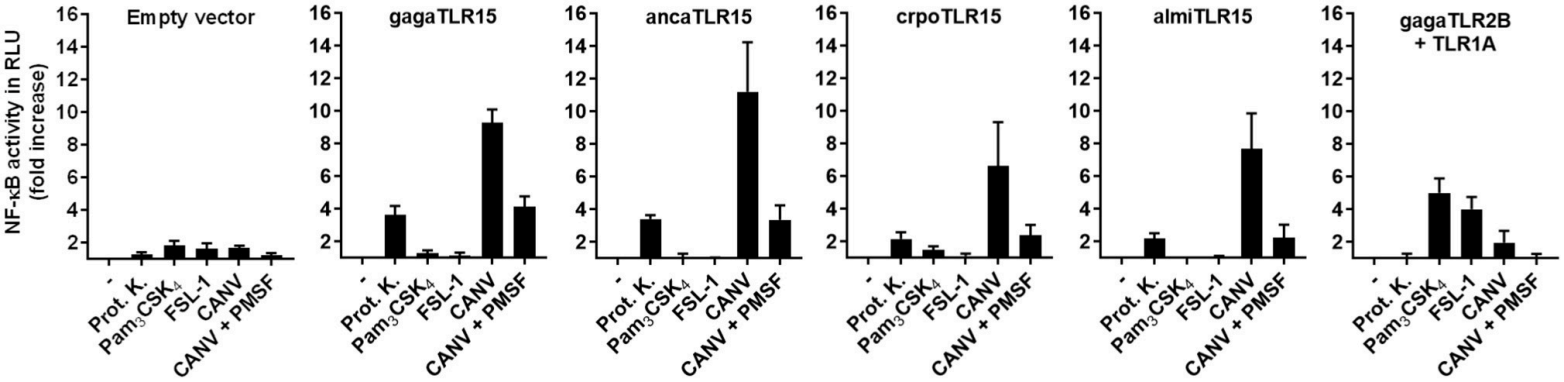
NF-κB activation by reptilian TLR15. HEK293 cells transiently transfected with an NF-κB luciferase reporter plasmid and empty vector, chicken (gaga), anolis (anca), crocodile (cpro), alligator (almi) TLR15 or the gagaTLR2B and gagaTLR1A plasmids were stimulated (5 h) with Proteinase K (100 ng/mL), Pam_3_CSK_4_ (100 ng/mL), FSL-1 (100 ng/mL), 10 μL of *Chrysosporium* anamorph of *Nannizziopsis vriesii* (CANV) sterile culture supernatant or 10 μL of CANV supernatant pre-treated (30 min) with 1 mM PMSF. Values are the mean ± SEM fold increase of NF-κB activity, represented by luciferase activity in Relative Light Units (RLU), in stimulated cells over unstimulated control cells from three independent experiments performed in duplicate.

Functionality of the cloned reptilian TLR15s was assessed after transfection of the plasmids encoding ancaTLR15, crpoTLR15, or almiTLR15, together with an NF-κB-luciferase reporter, into human HEK293 cells. Stimulation of reptilian TLR15-transfected cells with Proteinase K resulted in increased NF-κB activity (Figure 3), as was observed for the positive control cells transfected with gagaTLR15 (15). Reptilian and chicken TLR15 did not respond to the canonical TLR2/1 or TLR2/6 ligands Pam_3_CSK_4_ or FSL-1 resp. while these ligands were able to activate NF-κB in control cells that expressed the chicken TLR2B/TLR1A heterodimer (14). Previously, we and others found that gagaTLR15 is activated by secreted proteases of fungi pathogenic to poultry (15, 16). *Chrysosporium* anamorph of *Nannizziopsis vriesii* (CANV) is a pathogenic fungus which can cause a fatal condition called yellow fungus disease in infected reptiles (28). Use of culture supernatant of a clinical CANV isolate in our stimulation assay potently activated NF-κB in cells expressing reptilian or chicken TLR15 but not chicken TLR2B/TLR1A. Addition of the serine protease inhibitor PMSF to the CANV culture supernatant strongly reduced its TLR15 activating capacity, confirming the response of TLR15 to proteolytic activity (Figure 3). The responsiveness of the newly identified TLR15 (previously erroneously annotated as TLR2) of *A. carolinensis, C. porosus*, and *A. mississippiensis* indicates that these receptors are indeed still functional and share functional characteristics with chicken TLR15 and not chicken TLR2 or TLR1.

### Proteolytic cleavage and variable expression of TLR15

**Figure 4:**
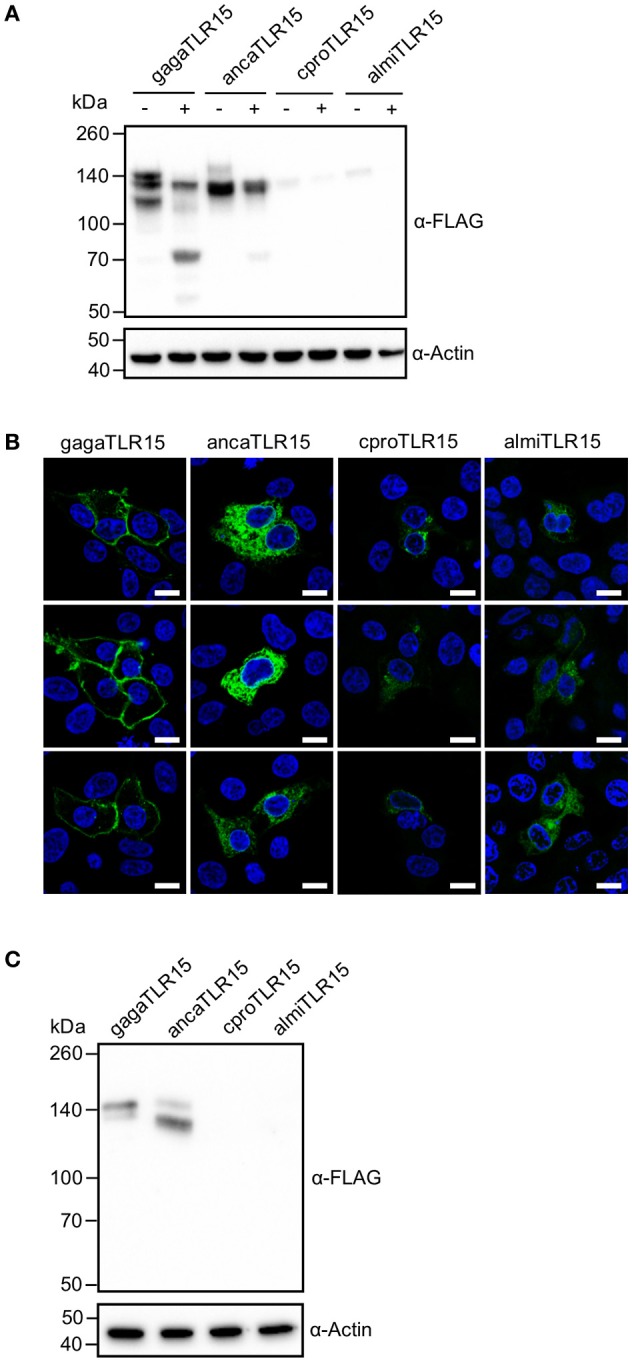
Proteolytic cleavage and expression of reptilian TLR15. **(A)** Immunoblot analysis of HEK293 cells expressing C-terminally FLAG-tagged chicken (gaga), anolis (anca), crocodile (cpro), or alligator (almi) TLR15 left untreated (–) or stimulated (+) (1 h) with 250 ng/mL Proteinase K. Mature TLR15 is ~140 kDa. Treatment with Proteinase K results in cleavage of gagaTLR15 and ancaTLR15 to form a cleaved receptor fragment that is slightly higher than 70 kDa. Note that crpoTLR15 and almiTLR15 are poorly expressed compared to ancaTLR15 and gagaTLR15. Beta-actin was detected to confirm equal loading of total protein onto SDS-PAGE gel. **(B)** Confocal microscopy on HEK293 cells expressing C-terminally HA-tagged TLR15 (green). Note that crpoTLR15 and almiTLR15 show lower expression compared to ancaTLR15 and gagaTLR15. All images were produced with the same microscopy settings. Nuclei are stained with DAPI (blue). White scale bar is 10 μm. Three representative images from two independent experiments are shown for each transfected group. **(C)** Immunoblot analysis of reptilian viper heart (VH-2) cells transfected with the different FLAG-tagged TLR15s. The rabbit α-human Beta actin antibody cross reacts with a specific protein in VH-2 cell lysate which was used to confirm equal loading of total protein onto SDS-PAGE gel. For **(A,C)**; results are representative of three independent experiments.

To ensure that the protease treatment of cells transfected with reptilian TLR15 resulted in proteolytic cleavage of the receptor, cells expressing a C-terminal FLAG-tagged TLR15 were incubated with Proteinase K, lysed and subjected to Western blotting using a FLAG-specific antibody. Proteinase K cleaved ancaTLR15 to form a similarly sized product as gagaTLR15 (slightly higher than 70 kDa) (15), yet the efficiency of cleavage of ancaTLR15 was substantially less than noted for gagaTLR15 (Figure 4A). Although both crpoTLR15 and almiTLR15 have a similar molecular size as gagaTLR15 and ancaTLR15, no cleaved forms of these receptors were detected; however, this may be due to the generally low level of expression of these receptors in whole cell lysates (Figure 4A). Detection of the various TLR15 receptors using confocal microscopy showed that HEK293 cells transfected with gagaTLR15 strongly expressed this receptor at the cell surface, in line with previous findings in different cell-lines (15). Detection of the ancaTLR15 also indicated strong expression but this TLR resided mostly intracellularly (Figure 4B). Interestingly, despite a higher protein similarity to gagaTLR15 than to ancaTLR15, both crocodilian TLR15s localized mostly intracellularly but with low signal intensity (Figure 4B), consistent with the observed low protein expression levels for these receptors (Figure 4A). In an attempt to improve expression of the crocodilian receptors we transfected plasmids into viper heart (VH-2) reptilian cells rather than human HEK293 cells. In the reptilian cells the crpoTLR15 and almiTLR15 proteins still could not be detected by Western blotting in contrast to ancaTLR15 and gagaTLR15 (Figure 4C). These findings suggest that the different TLR15s display a species-specific difference in protein expression efficiency.

### Species-specific codon bias of TLR15

**Figure 5:**
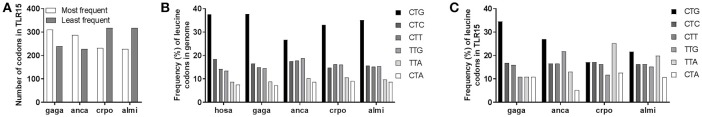
Species-specific bias in TLR15 codon usage. **(A)** The number of most frequent or least frequent codons in chicken (gaga), anolis (anca), crocodile (cpro), and alligator (almi) TLR15 according to codon prevalence in the human genome (see also Supplementary Table 3). **(B)** Frequency of the six codons encoding the amino acid leucine in the genomes of *Homo sapiens* (hosa), *Gallus gallus* (gaga), *Anolis carolinensis* (anca), *Crocodylus porosus* (crpo), and *Alligator mississippiensis* (almi). **(C)** Frequency of leucine codons in TLR15 of the indicated species.

In search for the molecular basis of the variable expression levels of the different TLR15s in human and reptilian cells, we compared the codon usage of the different *tlr15* genes. Codon usage bias, the organism-specific use of different synonymous codons to encode the same amino acid, is well-known for its effect on heterologous protein expression efficiency. To investigate whether the limited expression of crpoTLR15 and almiTLR15 protein in human cells could be due to codon bias, we analyzed codon usage of the four *tlr15* genes in relation to the genome-wide codon usage in human (Supplementary Table 3). While gaga*tlr15* and anca*tlr15* contain more frequently than infrequently used codons, the opposite was found for crpo*tlr15* and almi*tlr15* (Figure 5A). The higher number of infrequent codons in crocodile and alligator *tlr15* transcripts may reduce translation efficiency of these receptors resulting in lower protein expression levels compared to chicken and anolis TLR15.

**Figure 6:**
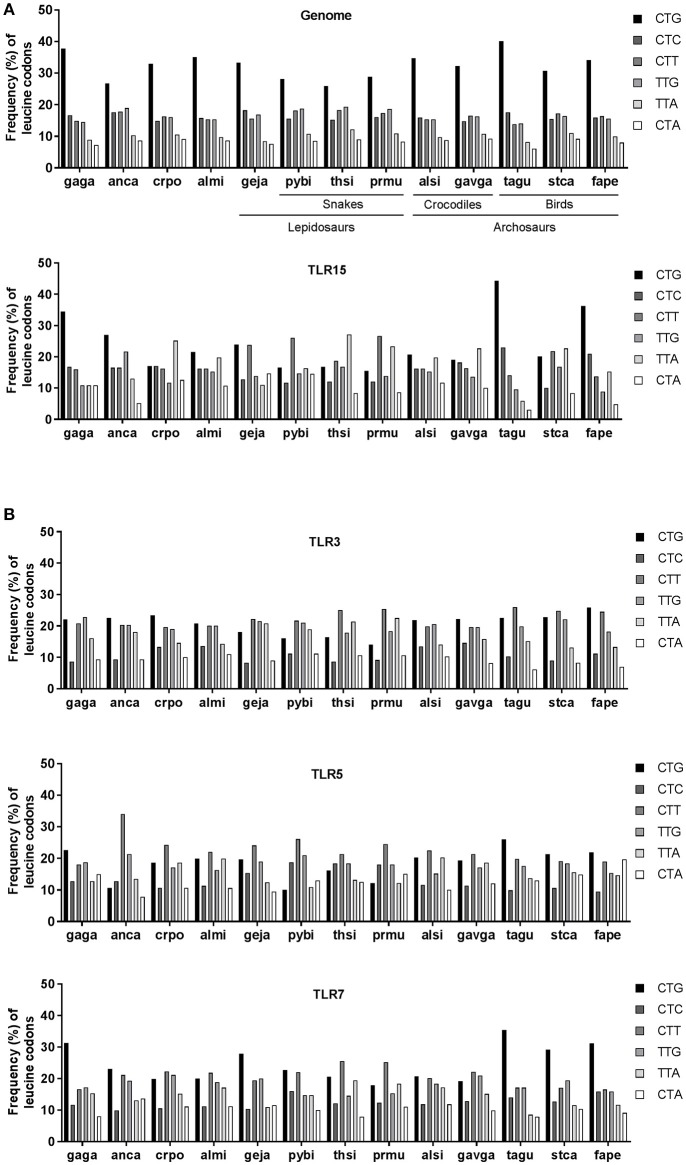
Leucine codon usage in TLRs of lepido- and archosaurians. **(A)** Frequency (percentage) of the six codons encoding leucine across genome-wide coding sequences (upper panel) or in TLR15 (lower panel) in lepidosaurians and archosaurians. **(B)** Frequency of leucine codons in TLR3, TLR5, and TLR7 in lepidosaurians and archosaurians. Species abbreviations: gaga (*Gallus gallus*, bird), anca (*Anolis carolinensis*, lizard), crpo (*Crocodylus porosus*, crocodile), almi (*Alligator mississippiensis*, crocodile), geja (*Gekko japonicus*, lizard), pybi (*Python bivittatus*, snake), thsi (*Thamnophis sirtalis*, snake), prmu (*Protobothrops mucrosquamatus*, snake), alsi (*Alligator sinensis*, crocodile), gavga (*Gavialis gangeticus*, crocodile), tagu (*Taeniopygia guttata*, bird), stca (*Struthio camelus australis*, bird), fape (*Falco peregrinus*, bird).

As codon usage may differ between species, we next compared human, chicken, anolis, crocodile, and alligator genome-wide usage of leucine codons. We focused on leucine as this amino acid can be encoded by six codons and is the most abundant amino acid in TLRs, including TLR15. Results showed that genome-wide leucine codon usage is conserved among human, chicken, anolis, crocodile and alligator and that in all species the CTG leucine codon is most abundant and CTA and TTA are least abundant (Figure 5B). In clear contrast to genome-wide usage of leucine codons, leucine codon usage in *tlr15* genes is markedly different between species. For example, CTG codon usage in gagaTLR15 is 34% (41/119) vs. only 17% in crpoTLR15 (19/111) while TTA codon usage is just 11% in gagaTLR15 (13/119) but 25% in crpoTLR15 (28/111). The latter is more than twice the average of TTA codon usage in both the chicken and crocodile genome (Figure 5C). Additional analysis of the other TLR15 sequences identified within the phylogenetic tree also showed extensive variation in leucine codon usage among lepido- and archosaurians despite similar genome-wide leucine codon usage in these species (Figure 6A). Interestingly, the same analysis of TLR3, TLR5, and TLR7, which unlike TLR15 are highly conserved among vertebrate species, revealed a more conserved pattern of leucine codon usage among the same set of species, especially in the case of TLR3 (Figure 6B). These findings indicate that TLR15, which has been lost in most vertebrates, shows a species-specific bias of leucine codon usage with greater interspecies variability than TLRs that have been conserved across most vertebrates.

**Figure 7:**
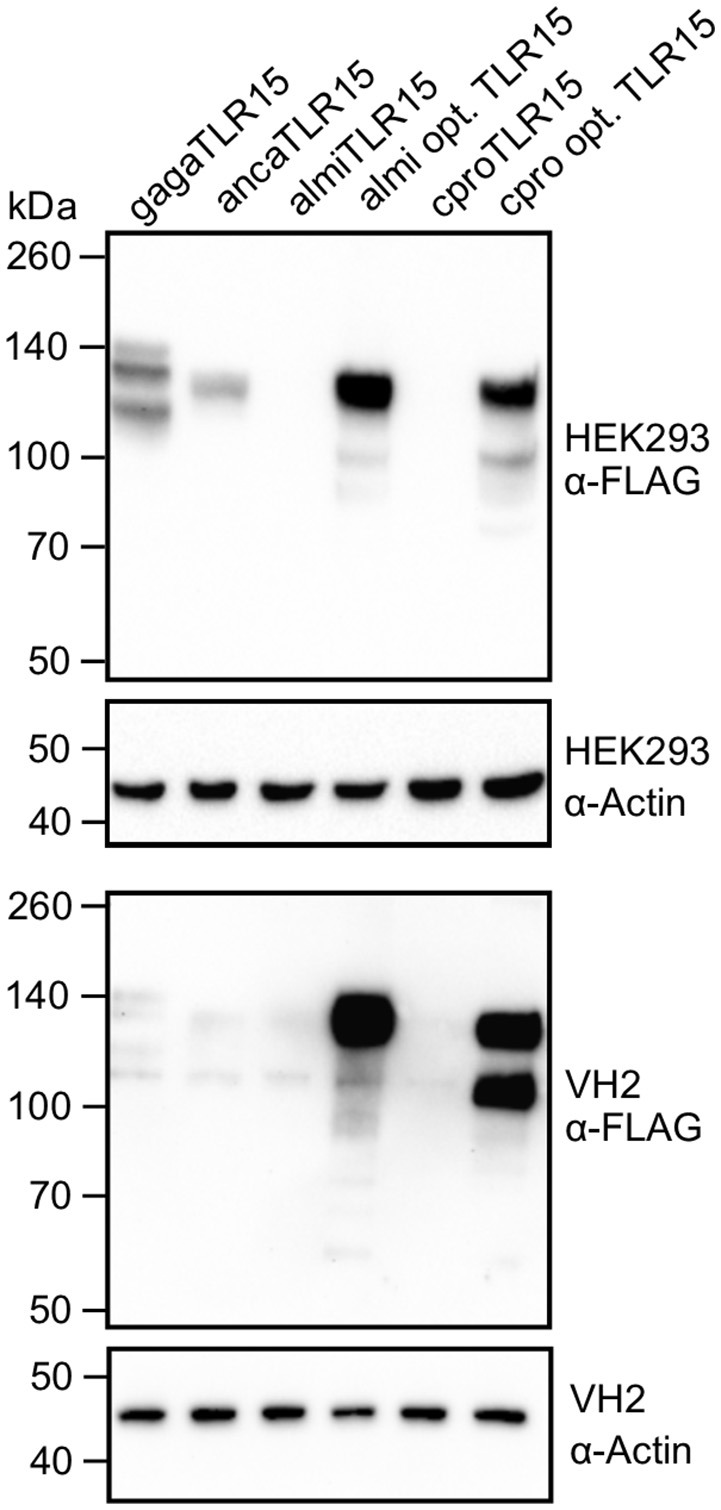
Expression of codon optimized alligator and crocodile TLR15. Immunoblot analyses of HEK293 cells and VH-2 cells expressing C-terminal FLAG-tagged chicken (gaga), anolis (anca), alligator (almi), codon optimized alligator (almi opt.), crocodile (cpro), or codon optimized crocodile (crpo opt.) TLR15. Mature TLR15 is ~140 kDa. Beta-actin was detected to confirm equal loading of HEK293 and VH-2 total protein onto SDS-PAGE gel. Results are representative of at least two independent experiments.

To verify that the identified species-specific codon usage in *tlr15* genes is a major cause of the observed variable expression levels of TLR15, we transfected HEK293 and VH-2 cells with synthetic alligator and crocodile *tlr15* genes that had been codon optimized according to human codon usage. For leucine residues in almiTLR15 and crpoTLR15 the optimization resulted in ≤ 5% of leucines being encoded by the CTC codon and ≥ 95% being encoded by the preferred CTG codon. Transfection of the codon optimized genes resulted in very high expression of both TLR15 proteins in human HEK293 cells as well as in reptilian VH-2 cells (Figure 7). This clearly indicates that gene-specific evolutionary changes of codon usage have a major impact on relative protein expression efficiency, including the expression of reptilian TLR15.

## Discussion

Throughout evolution, duplications, and losses of TLR genes have resulted in varying repertoires of TLRs among animal lineages. While some TLRs are highly conserved in nearly all vertebrates, other TLRs, most notably members of the TLR1 subfamily, evolved much more dynamically and are occasionally only found in specific vertebrate lineages. In the present work we provide evidence that (i) TLR15 is evolutionarily older than expected, (ii) *tlr15* genes display species-specific codon usage, (iii) the *tlr15* gene underwent evolutionary regression in most vertebrates including certain reptilian lineages, and (iv) that the activation of reptilian TLR15 by external proteases is a conserved feature that functionally distinguishes TLR15 from other TLR1 subfamily members. TLR15 was originally identified in chickens (17) and later studies found additional TLR15 orthologs in other avian and also four reptilian species (green anole lizard, Burmese python, Chinese alligator, and American alligator) (6, 16, 29). By combining phylogenetic and synteny analyses results, we identified TLR15 orthologs in even more reptilian species. All of the newly identified *tlr15* genes in lepidosaurians and archosaurians (including avian *tlr15* genes) are erroneously annotated in the database as TLR1 or TLR2 sequences. However, based on their phylogeny as well as highly conserved position in the genome and, most convincingly, the activation of anolis (lepidosaurian), salt water crocodile and alligator (archosaurians) TLR15 by proteolytic cleavage, we consider these sequences as bonafide TLR15 orthologs.

Unexpectedly, our bioinformatics search of a wide range of vertebrate genomes also led to the identification of a TLR sequence in the genome of the Australian ghost shark that has high homology to, and the same synteny as, avian and reptilian TLR15. The presence of this putative TLR15 ortholog in a shark species suggests that TLR15 did not arise in the sauropsid lineage but instead originates before the divergence of chondrichthyes fish and tetrapods. This would date the origin of the ancestral TLR15 to at least 465 million years ago (30) while reptiles share a common ancestor with birds roughly 284 million years ago (30, 31).

The successful expression of recombinant reptilian TLR15s in human cells allowed us to perform functional studies. NF-κB reporter assays with TLR15-transfected cells clearly showed that the cloned reptilian TLR15 was proteolytically cleaved and activated by fungal proteases, as has previously been reported for chicken TLR15 (15, 16). Chicken and reptilian TLR15 are predicted to share many structural characteristics including a highly conserved region in LRR11 which may be involved in the TLR15 activation process (29).

Given that both crocodilian TLR15 protein sequences are more similar to chicken than to anolis TLR15, we were surprised to find highly variable expression levels between the different TLR15s. Western blot analysis and confocal microscopy indicated high expression of gagaTLR15 and ancaTLR15 in a human and reptilian cell-line while crpoTLR15 and almiTLR15 protein levels were much lower. Investigation of *tlr15* codon usage pointed to a potential molecular basis for this difference in protein expression. The analysis revealed that both crocodilian *tlr15* genes are biased more toward using unpreferred codons than the *tlr15* genes of chicken and anolis. This was especially true for codons for leucine, the most abundant amino acid in TLR15. Codon optimization of both crocodile *tlr15* genes resulted in strongly increased protein levels, indicating that codon bias is an important determinant of TLR15 expression. Practically, these findings demonstrate that codon usage is a significant factor to consider when designing experiments to study TLRs in heterologous expression systems. The biological rationale for the use of unpreferred codons by crocodilian and some other reptilian TLR15s remains to be clarified. We found higher variability in leucine codon usage among TLR15s compared to TLRs that are more conserved among vertebrates such as TLR3, indicating that variation in leucine codon bias is not a general feature of TLR evolution following speciation, but is perhaps more related to the biological role of TLR15. Zhong et al. first described that most human *tlr* genes are not enriched with preferred codons and that this considerably limits TLR expression (32). Codon bias in mammalian *tlr7* leading to low cytosine-guanine (CG) content was shown to limit *tlr7* transcription and this has been proposed to form a regulatory mechanism to prevent over-expression of TLR7 which can lead to auto-immune disease (33). Thus, some TLRs may have become biased in codon usage under a selective pressure to maintain suboptimal codons that limit their expression efficiency. However, we identified variation in the biased usage of leucine codons in TLR15 among reptiles. While crocodilian and snake TLR15s are biased to contain more unpreferred leucine codons, most bird and lizard TLR15s contain predominantly preferred leucine codons. Additionally, the biased leucine codon usage among the three relatively close related snake species is inconsistent (Figure 6A). Given this diversity in TLR15 leucine codon usage, which is not in line with the evolutionary relations among these species, it seems likely that the species-specific TLR15 codon bias is more the result of neutral mutation and drift than of selection. Evolution of codon bias through neutral mutation and drift is common for most genes in higher eukaryotes (34–37). From the perspective of immune system evolution, it is noteworthy that the variable leucine codon usage among TLR15s coincides with large scale loss of TLR15 from the teleost fish, amphibian, and mammalian lineages. Perhaps even more striking are the identified TLR15-like remnants or complete absence of a TLR15-like sequence in turtles which are genetically closely related to crocodiles and birds (18). These independent gene loss events in different animal lineages and even among reptiles, suggest multiple moments of redundancy of TLR15 throughout vertebrate evolution. It is possible that in species that lost TLR15, other receptors for microbial proteases have taken over its role (38–40). In other words, it can be speculated that species-specific codon usage and the persistence of unpreferred codons are part of the onset to gradual functional redundancy and eventually disappearance of TLR15 from a genome, but this awaits detailed analysis of TLR codon usage in relation to the evolutionary history of TLRs across various vertebrate lineages.

## Methods and materials

### Isolation of reptilian DNA and ethics statement

Whole blood from *C. porosus* and *A. mississippiensis* was collected via the spinal vein (41) with an 18 ga needle and a 3 mL syringe. Blood was immediately transferred to a 4 mL heparin Vaccutainer^TM^, and 200 μL was centrifuged at 2,500 × g for 5 min. DNA was isolated from the resulting cell pellet using a Qiamp® DSP DNA kit (Qiagen). Genomic DNA was precipitated with 3 M NaOAc in the presence of 70% isopropyl alcohol. Precipitated DNA was washed with 70% EtOH and resuspended in nuclease free water. All procedures related to the handling of crocodilians were conducted as approved by the McNeese State University Animal Care and Use Committee. Genomic DNA of *A. carolinensis* was isolated as described (23). The procedure was approved by the Animal Ethics Committee of Utrecht University (study number 2014.II.04.031).

### Plasmid constructs

Phusion high-fidelity DNA polymerase, dNTPs, fast digest restriction endonucleases, T4 DNA ligase, and primers were purchased from Thermo Fisher Scientific. The TLR15 gene of *A. carolinensis* was amplified from genomic DNA by touchdown PCR with gene-specific primers listed in Supplementary Table 4. The purified ancaTLR15 gene was next amplified to add a KpnI restriction and kozak site at the 5′ end of the gene and an overlap on a 3 × Hemagglutinin-epitope (HA) sequence on the 3′ end of the gene. A 3 × HA sequence was amplified from a pTracer-CMV2ΔGFP/3 × HA vector (42) to add an overlap to ancaTLR15 at the start of the HA sequence and a PacI restriction site at the end of the sequence. The ancaTLR15 gene and 3 × HA sequence were subsequently fused by standard overlap PCR, digested with KpnI and PacI and ligated in pTracer-CMV2ΔGFP to yield ancaTLR15 carrying a C-terminal 3 × HA-tag. TLR15 genes of *C. porosus* and *A. mississippiensis* were amplified from genomic DNA by touchdown PCR (for primers see Supplementary Table 4), digested with KpnI and NotI and ligated into pTracer-CMV2ΔGFP/3 × HA (from which ancaTLR15 was removed) or a vector with a 3 × FLAG epitope tag [pTracer-CMV2ΔGFP/3 × FLAG, (43)] to yield crpoTLR15 or almiTLR15 with C-terminal 3 × HA-tag or 3 × FLAG-tag. The gagaTLR15 gene was cut from gagaTLR15-pTracer (15) with KpnI and NotI and ligated into pTracer-CMV2ΔGFP/3 × HA or pTracer-CMV2ΔGFP/3 × FLAG to yield gagaTLR15 with C-terminal 3 × HA-tag or 3 × FLAG-tag. The codon optimized *A. mississippiensis* and *C. porosus* TLR15 genes were synthesized by GeneArt (Thermo Fisher Scientific) and subcloned using KpnI and NotI restriction sites into pTracer-CMV2ΔGFP/3 × FLAG. All constructs were verified by sequencing (Macrogen). TLR15 sequences were deposited in GenBank with the following accession numbers: anca*tlr15* (MH395322), crpo*tlr15* (MH395323), almi*tlr15* (MH395324).

### Phylogenetic and synteny analysis

Protein sequences of the TLR1 subfamily of multiple vertebrate species including actinopterygii, sarcopterygii, chondrichtyes, amphibia, mammalia, and reptilia (lepidosaurs and archosaurs, including aves) (Supplementary Table 1), were identified by BLASTp on the species' ref_seq database of the NCBI using the TIR domain of TLRs from reference species (anole, chicken, human, zebrafish, xenopus) as queries. Full length sequences were collected in FASTA format. When multiple copies of the same annotated TLR (i.e., duplications) were found in the one species, receptors were denoted as TLR2, TLR2-1, TLR2-2 etc. Sequences were aligned with the MUltiple Sequence Comparison by Log- Expectation (MUSCLE) sequence aligner of the EMBL-EBI (https://www.ebi.ac.uk/Tools/msa/muscle/). MEGA7 software (25) was used to construct a phylogenetic tree from the aligned TLR sequences by Maximum Likelihood analysis. The analysis involved 136 amino acid sequences. All positions containing gaps and missing data were eliminated. There were a total of 252 positions in the final dataset. The best fitting substitution model was JTT+G+I (AICc: 65408,13971; BIC: 67720,59789; Gamma: 1,275251655, Invariant: 0,033209398). Two-hundred and fifty bootstrap iterations were run and the tree with the highest log likelihood was exported in Newick format and customized in iTol (https://itol.embl.de) (26). The genomic region surrounding the *tlr15* locus was inspected for neighboring genes by searching the NCBI Gene database with the gagaTLR15 gene ID or the gene ID of predicted TLR1 or 2 sequences of species clustering with gagaTLR15 in the phylogenetic tree. For species in which no TLR15 ortholog was found, the region between *erlec1* and *gpr75* was analyzed by using BLASTx on NCBIs non-redundant protein sequence database (NR) of *Gallus gallus* (taxid: 9031). Database searches were performed in April 2018.

### Protein sequence and codon analyses

Protein sequences were aligned using the Clustal Omega sequence aligner (https://www.ebi.ac.uk/Tools/msa/clustalo/) (44). To identify the TIR domain (45) the secondary structure was predicted using Jpred4 (http://www.compbio.dundee.ac.uk/jpred/) (46). Transmembrane region was predicted with http://www.cbs.dtu.dk/services/TMHMM/ (47). LRRs were identified by manual sequence inspection according to Matsushima et al. (27) and with use of the Leucine rich repeat finder web tool http://www.lrrfinder.com/ (48). Signal peptides were predicted with http://www.cbs.dtu.dk/services/SignalP/ (49). Codon usage tables of the different species were retrieved from https://hive.biochemistry.gwu.edu/cuts/about (50).

### Cell culture and transient transfection

HEK293 cells were cultured in DMEM (Thermo Fisher Scientific) supplemented with 5% FCS (Bodinco) at 37°C and 10% CO_2_. Viper heart (VH-2) cells from a Russell's viper (*Daboia russelli*) were cultured in M199 (Thermo Fisher Scientific) with Hank salts and 10% FCS at 31°C in air. Cells were transiently transfected at 70% confluency with Fugene HD (Promega) at a DNA to Fugene ratio of 1:3 according to the manufacturer's instructions.

### Fungal supernatant

*Chrysosporium* anamorph of *Nannizziopsis vriesii* (CANV) isolated from an agama (lizard) patient was kindly provided by the Veterinary Microbiological Diagnostics Center (VMDC) of the Utrecht University. CANV was grown in 25 mL M199 liquid medium for 7 days at 26°C. Supernatant was collected by centrifugation [3,000 × g, 5 min, room temperature (RT)] and sterilized by passaging through a 0.2 μm filter. Supernatant was stored at 4°C until use (within 24-h). Prior to addition to cells, CANV supernatant was treated with 1 mM of phenylmethane sulfonyl fluoride (PMSF) for 30 min at RT.

### Luciferase NF-κB reporter assay

Cells were transfected in a 12-well plate with 50 ng of an NF-κB-luciferase reporter plasmid and 450 ng of HA-tagged TLR15 plasmid or 225 ng of gagaTLR2B and 225 ng of gagaTLR1A plasmid (14). Twenty-four hours after transfection cells were redistributed into a 96-well plate. After 24-h cells were washed twice with DMEM without FCS and stimulated with: 100 ng/mL of Proteinase K (Sigma), Pam_3_CSK_4_, FSL-1 (both Invivogen) or 10 μL PMSF treated or untreated CANV culture supernatant in a total of 100 μL DMEM without FCS. After 5 h at 37°C cells were lysed in 50 μL reporter lysis buffer (Promega) at −80°C for 24-h. After thawing lysate was mixed with luciferase reagent (Promega) and luciferase activity was measured in a TriStar2 luminometer (Berthold). NF-κB activity is represented by luciferase activity in Relative Light Units (RLU). Results were expressed as fold increase in NF-κB activity of stimulated over unstimulated cells.

### Confocal microscopy

Cells were transfected in a 12-well plate with 500 ng of HA-tagged TLR15 plasmid. Glass coverslips were coated overnight with 0.02% Poly-L-lysine (Sigma) at RT. Coverslips were washed three times with PBS (Sigma) and 24-h after transfection cells were seeded onto coated coverslips. Twenty-four hours after seeding onto coverslips cells were washed once with TRIS-buffered saline (TBS) and fixed with TBS/1.5% paraformaldehyde (Affimetrix). Cells were permeabilized and blocked (30 min) with TBS containing 0.1% saponin and 0.2% BSA (both Sigma). Next, cells were incubated (1 h) with Alexa Fluor-488 conjugated mouse α-HA antibody (A21287; Thermo Fisher Scientific) and DAPI (Molecular Probe). After staining cells were washed with TBS and MilliQ and embedded in Prolong Diamond mounting solution (Thermo Fisher Scientific). Cells were imaged on a Leica SPE-II laser confocal microscope and images were processed with Leica LAS AF software.

### TLR15 expression and cleavage

HEK293 and VH-2 cells were transfected in a 6-well plate with 1,000 ng of FLAG-tagged TLR15 plasmids. After 48-h HEK293 cells were washed with DMEM and incubated (1 h, 37°C) with 250 ng/mL Proteinase K in DMEM without FCS. HEK293 and VH-2 cells were lysed in lysis buffer [25 mM TRIS, 150 mM NaCl, 0.5% NP-40, 1 mM EDTA, 5% glycerol, 1 cOmplete protease inhibitor tablet (Roche)], centrifuged (3,000 × g, 3 min, RT) and total protein concentration in the supernatant was measured by BCA assay (Thermo Fisher Scientific). Samples were equalized and run on SDS-PAGE gel and blotted onto PVDF membrane. Membranes were blocked with 5% non-fat milk in TBS-Tween (0.1%) and incubated (1 h, RT) with M2 mouse α-FLAG antibody (F3165; Sigma) or rabbit α-human Beta actin (bs-0061R; Bioss) followed by incubation (1 h, RT) with goat α-mouse (A2304; Sigma) or goat α-rabbit (A4914; Sigma) HRP conjugated antibody. HRP chemiluminescence was detected with Clarity western ECL (Bio-rad). The rabbit α-human Beta actin cross reacts with a specific protein in VH-2 cell lysate which is likely actin of *D. russelli* due to very high evolutionary conservation of actin.

## Author contributions

CV, JW, and JvP: designed research; MM and CV: isolated reptilian DNA; CV: performed research, analyzed data, and wrote manuscript with support from all authors.

### Conflict of interest statement

The authors declare that the research was conducted in the absence of any commercial or financial relationships that could be construed as a potential conflict of interest.

## References

[1] Medzhitov R. Toll-like receptors and innate immunity. Nat Rev Immunol. (2001) 1:135–45. 10.1038/35100529 11905821

[2] Gay NJ Gangloff M. Structure and function of Toll receptors and their ligands. Annu Rev Biochem. (2007) 76:141–65. 10.1146/annurev.biochem.76.060305.151318 17362201

[3] Leulier F Lemaitre B. Toll-like receptors–taking an evolutionary approach. Nat Rev Gen. (2008) 9:165–78. 10.1038/nrg2303 18227810

[4] Roach JC Glusman G Rowen L Kaur A Purcell MK Smith KD. The evolution of vertebrate Toll-like receptors. Proc Natl Acad Sci USA. (2005) 102:9577–82. 10.1073/pnas.0502272102 15976025 PMC1172252

[5] Rauta PR Samanta M Dash HR Nayak B Das S. Toll-like receptors (TLRs) in aquatic animals: signaling pathways, expressions and immune responses. Immunol Lett. (2014) 158:14–24. 10.1016/j.imlet.2013.11.013 24291116

[6] Alcaide M Edwards SV. Molecular evolution of the Toll-like receptor multigene family in birds. Mol Biol Evol. (2011) 28:1703–15. 10.1093/molbev/msq351 21239391

[7] Jin MS Kim SE Heo JY Lee ME Kim HM Paik S-G. Crystal structure of the TLR1-TLR2 heterodimer induced by binding of a tri-acylated lipopeptide. Cell (2007) 130:1071–82. 10.1016/j.cell.2007.09.008 17889651

[8] Kang JY Nan X Jin MS Youn S-J Ryu YH Mah S. Recognition of lipopeptide patterns by Toll-like receptor 2-Toll-like receptor 6 heterodimer. Immunity (2009) 31:873–84. 10.1016/j.immuni.2009.09.018 19931471

[9] Kiura K Kataoka H Nakata T Into T Yasuda M Akira S. The synthetic analogue of mycoplasmal lipoprotein FSL-1 induces dendritic cell maturation through Toll-like receptor 2. FEMS Immunol Med Microbiol. 46:78–84. 10.1111/j.1574-695X.2005.00002.x 16420600

[10] Kruithof EK Satta N Liu JW Dunoyer-Geindre S Fish RJ. Gene conversion limits divergence of mammalian TLR1 and TLR6. BMC Evol Biol. (2007) 7:148. 10.1186/1471-2148-7-148 17727694 PMC2077338

[11] Fink IR Pietretti D Voogdt CGP Westphal AH Savelkoul HFJ Forlenza M. Molecular and functional characterization of Toll-like receptor (Tlr)1 and Tlr2 in common carp (*Cyprinus carpio*). Fish Shellfish Immunol. (2016) 56:70–83. 10.1016/j.fsi.2016.06.049 27368535

[12] Solbakken MH Tørresen OK Nederbragt AJ Seppola M Gregers TF Jakobsen KS. Evolutionary redesign of the Atlantic cod (*Gadus morhua* L.) Toll-like receptor repertoire by gene losses and expansions. Sci Rep. (2016) 6:25211. 10.1038/srep25211 27126702 PMC4850435

[13] Velová H Gutowska-Ding MW Burt DW Vinkler M Toll-like receptor evolution in birds: gene duplication, pseudogenisation and diversifying selection. Mol Biol Evol. (2018). 35:2170–84. 10.1093/molbev/msy119 PMC6107061 29893911

[14] Keestra AM de Zoete MR van Aubel RA van Putten JP. The central leucine-rich repeat region of chicken TLR16 dictates unique ligand specificity and species-specific interaction with TLR2. J Immunol. (2007) 178:7110–9. 10.4049/jimmunol.178.11.7110 17513760

[15] de Zoete MR Bouwman LI Keestra AM van Putten JPM. Cleavage and activation of a Toll-like receptor by microbial proteases. Proc Natl Acad Sci USA. (2011) 108:4968–73. 10.1073/pnas.1018135108 21383168 PMC3064367

[16] Boyd AC Peroval MY Hammond JA Prickett MD Young JR Smith AL. TLR15 Is unique to avian and reptilian lineages and recognizes a yeast-derived agonist. J Immunol. (2012) 189:4930–8. 10.4049/jimmunol.1101790 23066147

[17] Higgs R Cormican P Cahalane S Allan B Lloyd AT Meade K. Induction of a novel chicken Toll-like receptor following *Salmonella enterica* serovar Typhimurium infection. Infect Immun. (2006) 74:1692–8. 10.1128/IAI.74.3.1692-1698.2006 16495540 PMC1418683

[18] Wang Z Pascual-Anaya J Zadissa A Li W Niimura Y Huang Z. The draft genomes of soft-shell turtle and green sea turtle yield insights into the development and evolution of the turtle-specific body plan. Nat Genet. (2013) 45:701–6. 10.1038/ng.2615 23624526 PMC4000948

[19] Zimmerman LM Vogel LA Bowden RM. Understanding the vertebrate immune system: insights from the reptilian perspective. J Exp Biol. (2010) 213:661–71. 10.1242/jeb.038315 20154181

[20] Merchant M Fleury L Rutherford R Paulissen M. Effects of bacterial lipopolysaccharide on thermoregulation in green anole lizards (*Anolis carolinensis*). Vet Immunol Immunopathol. (2008) 125:176–81. 10.1016/j.vetimm.2008.04.014 18514328

[21] Merchant M Williams S Trosclair PL III Elsey RM Mills K. Febrile response to infection in the American alligator (*Alligator mississippiensis*). Comp Biochem Phys A (2007) 148:921–5. 10.1016/j.cbpa.2007.09.016 17977038

[22] Merchant ME Mills K Leger N Jerkins E Vliet KA McDaniel N. Comparisons of innate immune activity of all known living crocodylian species. Comp Biochem Phys B (2006) 143:133–7. 10.1016/j.cbpb.2005.10.005 16376129

[23] Voogdt CGP Bouwman LI Kik MJL Wagenaar JA van Putten JPM. Reptile Toll-like receptor 5 unveils adaptive evolution of bacterial flagellin recognition. Sci Rep. (2016) 6:19046. 10.1038/srep19046 26738735 PMC4703953

[24] Jones DT Taylor WR Thornton JM. The rapid generation of mutation data matrices from protein sequences. Comput Appl Biosci. (1992) 8:275–82. 1633570 10.1093/bioinformatics/8.3.275

[25] Kumar S Stecher G Tamura K. MEGA7: Molecular Evolutionary Genetics Analysis Version 7.0 for bigger datasets. Mol Biol Evol. (2016) 33:1870–4. 10.1093/molbev/msw054 27004904 PMC8210823

[26] Letunic I Bork P. Interactive tree of life (iTOL) v3: an online tool for the display and annotation of phylogenetic and other trees. Nucleic Acids Res. (2016) 44:W242–5. 10.1093/nar/gkw290 27095192 PMC4987883

[27] Matsushima N Tanaka T Enkhbayar P Mikami T Taga M Yamada K. Comparative sequence analysis of leucine-rich repeats (LRRs) within vertebrate toll-like receptors. BMC Genomics (2007) 8:124. 10.1186/1471-2164-8-124 17517123 PMC1899181

[28] Johnson RSP Sangster CR Sigler L Hambleton S Paré JA. Deep fungal dermatitis caused by the *Chrysosporium* anamorph of *Nannizziopsis vriesii* in captive coastal bearded dragons (*Pogona barbata*). Aust Vet J. (2011) 89:515–9. 10.1111/j.1751-0813.2011.00851.x 22103953

[29] Wang J Zhang Z Chang F Yin D. Bioinformatics analysis of the structural and evolutionary characteristics for toll-like receptor 15. PeerJ (2016) 4:e2079. 10.7717/peerj.2079 27257554 PMC4888287

[30] Hedges SB Marin J Suleski M Paymer M Kumar S. Tree of Life reveals clock-like speciation and diversification. Mol Biol Evol. (2015) 32:835–45. 10.1093/molbev/msv037 25739733 PMC4379413

[31] Benton MJ Donoghue PCJ. Paleontological evidence to date the tree of life. Mol Biol Evol. (2007) 24:26–53. 10.1093/molbev/msl150 17047029

[32] Zhong F Cao W Chan E Tay PN Cahya FF Zhang H. Deviation from major codons in the Toll-like receptor genes is associated with low Toll-like receptor expression. Immunology (2005) 114:83–93. 10.1111/j.1365-2567.2004.02007.x 15606798 PMC1782050

[33] Newman ZR Young JM Ingolia NT Barton GM. Differences in codon bias and GC content contribute to the balanced expression of TLR7 and TLR9. Proc Natl Acad Sci U.S.A. (2016) 113:E1362–71. 10.1073/pnas.1518976113 26903634 PMC4791032

[34] Chamary JV Parmley JL Hurst LD. Hearing silence: non-neutral evolution at synonymous sites in mammals. Nat Rev Gen. (2006) 7:98–108. 10.1038/nrg1770 16418745

[35] Hershberg R Petrov DA. Selection on codon bias. Annu Rev Genet. (2008) 42:287–99. 10.1146/annurev.genet.42.110807.091442 18983258

[36] Plotkin JB Kudla G Synonymous but not the same: the causes and consequences of codon bias. Nat Rev Gen. (2011) 12:32–42. 10.1038/nrg2899 PMC3074964 21102527

[37] Reis M dos Savva R Wernisch L. Solving the riddle of codon usage preferences: a test for translational selection. Nucleic Acids Res. (2004) 32:5036–44. 10.1093/nar/gkh834 15448185 PMC521650

[38] Ossovskaya VS Bunnett NW. Protease-activated receptors: contribution to physiology and disease. Physol Rev. (2004) 84:579–621. 10.1152/physrev.00028.2003 15044683

[39] Chavarría-Smith J Mitchell PS Ho AM Daugherty MD Vance RE. Functional and evolutionary analyses identify proteolysis as a general mechanism for NLRP1 inflammasome activation. PLOS Pathog. (2016) 12:e1006052. 10.1371/journal.ppat.1006052 27926929 PMC5142783

[40] LaRock CN Todd J LaRock DL Olson J O'Donoghue AJ Robertson AAB IL-1β is an innate immune sensor of microbial proteolysis. Sci Immunol. (2016) 1:eaah3539 10.1126/sciimmunol.aah3539 PMC5358671 28331908

[41] Zippel KC Lillywhite HB Mladinich CRJ. Anatomy of the crocodilian spinal vein. J Morphol. (2003) 258:327–35. 10.1002/jmor.10156 14584034

[42] Voogdt CGP Wagenaar JA van Putten JPM. Duplicated TLR5 of zebrafish functions as a heterodimeric receptor. Proc Natl Acad Sci USA. (2018) 115:E3221–9. 10.1073/pnas.1719245115 29555749 PMC5889648

[43] Keestra AM de Zoete MR Bouwman LI van Putten JPM. Chicken TLR21 is an innate CpG DNA receptor distinct from mammalian TLR9. J Immunol. (2010) 185:460–7. 10.4049/jimmunol.0901921 20498358

[44] Sievers F Wilm A Dineen D Gibson TJ Karplus K Li W. Fast, scalable generation of high-quality protein multiple sequence alignments using Clustal Omega. Mol Syst. Biol. (2011) 7:539. 10.1038/msb.2011.75 21988835 PMC3261699

[45] Xu Y Tao X Shen B Horng T Medzhitov R Manley JL. Structural basis for signal transduction by the Toll/interleukin-1 receptor domains. Nature (2000) 408:111–5. 10.1038/35040600 11081518

[46] Drozdetskiy A Cole C Procter J Barton GJ. JPred4: a protein secondary structure prediction server. Nucleic Acids Res. (2015) 43:W389–94. 10.1093/nar/gkv332 25883141 PMC4489285

[47] Krogh A Larsson B von Heijne G Sonnhammer ELL. Predicting transmembrane protein topology with a hidden Markov model: application to complete genomes. J Mol Biol. (2001) 305:567–80. 10.1006/jmbi.2000.4315 11152613

[48] Offord V Coffey TJ Werling D. LRRfinder: a web application for the identification of leucine-rich repeats and an integrative Toll-like receptor database. Dev Comp Immunol. (2010) 34:1035–41. 10.1016/j.dci.2010.05.004 20470819

[49] Petersen TN Brunak S von Heijne G Nielsen H. SignalP 4.0: discriminating signal peptides from transmembrane regions. Nat Methods (2011) 8:785–6. 10.1038/nmeth.1701 21959131

[50] Athey J Alexaki A Osipova E Rostovtsev A Santana-Quintero LV Katneni U. A new and updated resource for codon usage tables. BMC Bioinformatics (2017) 18:391. 10.1186/s12859-017-1793-7 28865429 PMC5581930

