# Supplementary material for: Evolutionary Regression and Species-Specific Codon Usage of TLR15
Source: Front Immunol. 2018 Nov 13;9:2626. doi: 10.3389/fimmu.2018.02626 (PMC6244663; doi:10.3389/fimmu.2018.02626)
Supplement: Supplementary file 1 [file Data_Sheet_1.PDF]

```
                Signal peptide
gagaTLR15   MGILIGSLYFYFISFLFSKVNGFLTQRTSPVSSFPFYNYSYLNLSSVSQAQAPKTARALN 60
crpoTLR15   MGILIRVLRFYLIAFLFNGANGFQTQRTSYMYGFKFSNYSYLNLSSIHEAQAPKTSRVLN 60
almiTLR15   MG--ILILRFYLIAFLFNGADGFQTQRTSHMYGFKFSNYSYLNLSSIHEAQAPKTSRVLN 58
ancaTLR15   MGTFIHSLHFCLIILSCHGESEFQNSETQIYVSKPSGH-HTFNYSSVTKQQTLQQS--LG 57
            **  *  * * :* :     . * ...*.   .    :   :* **: : *: : :  *.

             LRR1                    LRR2                     LRR3
gagaTLR15   FSYNAIEKITKRDFEGFHVLEVLDLSHNHIKDIEPGAFENLLSLVSVDLSFNDKNLLVSG 120
crpoTLR15   FSHNIIEKITRRDFEGFVALEVLDLSYNQIQDIEPGSFENLLSLVSVNLSFNDQLHRIPY 120
almiTLR15   FSHNVIEKITRRDFEGFVALEVLDLSYNQIQDIEPGAFENLLGLVSVNLSFNDHLHRIPY 118
ancaTLR15   SLQSTMKNVNKKMTDHSKTLESLNPLQNR------------SSI------LNS------- 92
              . ::::.::  :   .** *:   *:            .:      :*.

gagaTLR15   LAPHLKLIPTSGASGPSQIYMYFQKSAEAALEPSAPAELLPHLEDP----PNPGNVNPRF 176
crpoTLR15   LAPHLTFLQTGEASGIPQHNIYFERSSEAALESFVSAEEQQYPEGL----YGLVNSHSKF 176
almiTLR15   LAPHLTFLQTGETSGIPQHNIYFERSSEAALESFVSAEKQRYPEDS----HGLVNGHSKV 174
ancaTLR15   -------------TDYQVDELYTQKHTE-IMEVMDSSKDQPLLNARMSLEPRKHNLHEEP 138
                         :.     :* :: :*  :*    ::     :          * : .

                                                LRR4
gagaTLR15   R-----QRRTEENKTSPPAATLRPDLCGAPINGLLDLSRTKLSNEELTAKLDADLCQAQL 231
crpoTLR15   SRTARDLQEVDENRMVLPSPTLRHKICGKPINGVLDLSKSNLSEEELTEKMDASHCKDHL 236
almiTLR15   SRTVWDLQGVDENRTVFPSSTLRHKICGKPINGVLDLSKSNLSEEELTEKMDASHCKDHL 234
ancaTLR15   VLKLKAVDHRKHNDTPSPSVAMKPEGCGISMNGILDLSNRNLSEKELRAKIESGPCWATL 198
                      ..*    *: ::: . **  :**:****. :**::**  *:::. *   *

                LRR5                         LRR6
gagaTLR15   GTVLEFNISHSDLEMDLLSLFILFLPMKDIQSVDASYNRITINNIDVEAICHFPFSNFSF 291
crpoTLR15   DNIVELNISHNNLEIDLLSLFILLLPMENAQSIDASYNKITISNIELGKICDFPIRRLMF 296
almiTLR15   DSVVELNISHNNLEIDLLSLFILLLPMENAQSIDASYNKITISNIELGKICDFPIRRLLF 294
ancaTLR15   DKIQVLNASHNNLEGDLITLILLFLNMKNVRVIDLSCNNLTFNAMCAEEIQDLEESKLIF 258
            ..:  :* **.:** **::*::*:* *:: : :* * *.:*:. :    * .:   .: *

             LRR7                    LRR8                     LRR9
gagaTLR15   LNISNNPINSLETVCLPASITVIDLSFTNISTIPANFAKKLSKLERMYVQGNQLIYTVRP 351
crpoTLR15   VNISNNPLNSLDTVCLPSTIKIIDLSYTNINHIPKNFHEKLFNLERIYVQGNQFIYTVNS 356
almiTLR15   VNISNNPLNSLDTVCLPSTIKIIDLSYTNINHIPKNFHKKLFNLERIYVQGNQFIYTVNS 354
ancaTLR15   LNLSHNSLKTLSDLCLPQSLKGIDLSFTKIDRIPQEFAILFSNMEEIYLQGNQFVYTVKT 318
            :*:*:* :::*. :*** ::. ****:*:*. ** :*   : ::*.:*:****::***.

                                 LRR10                LRR11
gagaTLR15   ENPS-ATPRPPPGTVQISAISLVRNQAGTPIESLPESVKHLKVSNCSIVELPEWFANRMQ 410
crpoTLR15   DDSGKNVSKPKPGTVRITALSFVNTREGTPIESLPEKVKYLKMSNCSIVELPEWFARTMK 416
almiTLR15   DNSGKNVSKSQPGTVRIAALSLVNTREGTPIESLPEKVKYLKMSNCSIVELPEWFAHKMK 414
ancaTLR15   LQS------VLIGDVGTSSVSYVDLPKHSLIESLPHKVKHLVLSNCSIVELPEWFAQKVG 372
             :           * *  :::* *     : *****..**:* :*************. :

              LRR12                 LRR13                   LRR14
gagaTLR15   ELLFLDLSSNRISMLPDLPISLQQLDISNSDIKIIPPRFKSLSNLTVFNIQNNKLTEMHP 470
crpoTLR15   RLLFLDLSNNPISKLPDLPSSLQHLDLSNSDIKIIPPSFKSLANLTVFKIQSNKITDFSP 476
almiTLR15   KLLFLDLSNNPISKLPDLPSSLQHLDLSNSDIKIIPPSFKSLSNLTVFKIQSNKITDFSP 474
ancaTLR15   QLLFLDLSNNPMNSFPGLPTTLQRLDLSNSNIKAMA-NLKFISNLTVVNIPNNKIEDISP 431
            .*******.* :. :*.** :**:**:***:** :   :* ::****.:* .**: :: *

                   LRR15                  LRR16                 LRR17
gagaTLR15   EYFPSTLTTCDISKNKLKVLSLTKALENLESLNVSGNLITRLEPACQLPSLTNLDSSHNL 530
crpoTLR15   EYFLLTLTEYDVSKNKLKVLNLNENLRKAEYLNISGNVITQIDTTSPLSALTNLDSSHNL 536
almiTLR15   AYFLLTLTEYDVSKNKLKVLNLNENLRKAEFLNISGNVITQIDTTSPLSALTNLDGSHNL 534
ancaTLR15   KHVPYSLEEFDISKNKIRRMPFLGAHSKLKSLNISGNVIMQLNVNTSHPSLSNLDASHNL 491
             :.  :*   *:****:: : :     : : **:***:* :::      :*:***.****
```

```
                         LRR18                LRR19
gagaTLR15   ISELPDHLGQSLLMLKHFNLSGNKISFLQRGSLPASLEELDISDNAITTIVQDTFGQLTS 590
crpoTLR15   ISELPDHFAKFLPVLKYFNLSGNKISFLQPGSLPESLVELDISNNAITTIVEETFGHLTK 596
almiTLR15   ISELPDHFAEFLPVLKYFNLSGNKISFLQPGSLPESLVELDISNNAITTIVEETFGHLTK 594
ancaTLR15   ITELHDEMGTFLPELKFLNLSGNKISFLQPGSLPESLLELDISNNAITIIMEETFGRLRN 551
            *:** *.:.  *  **.:*********** **** ** *****:**** *:::***:* .

              LRR20                        CxCx24Cx20C  CTLRR
gagaTLR15   LSVLTVQGKHFFCNCDLYWFVNIYIRNPHLQINGKDDLRCSFPPDRRGSLVKSSNLTLLH 650
crpoTLR15   LNVLTVQGKHFFCNCDLYWFVNTYIHSPQLLINGRENLRCSFPTDKRGALVEKSNLTLVH 656
almiTLR15   LNVLTVQGKHFFCNCDLYWFVNTYIHSPQLLINGRESLRCSFPPDKRGALVEKSNLTLVH 654
ancaTLR15   LRVLMAQGKHFFCNCDLYWFANTYLASPNVQIHGREALKCSFPLQKRGLLVENSNLTILY 611
            * ** .**************.* *: .*:: *:*:: *:**** ::** **:.****:::

                        TM                                        TIR
gagaTLR15   CSLGIQMAITACMAILVVLVLTGLCWRFDGLWYVRMGWYWCMAKRRQYKKRPENKPFDAF 710
crpoTLR15   CSFGIQMAITACAAVLIMSVITSLCWHFDGPWYIRMGWYWCMAKRRQYEKRPENKTYDAF 716
almiTLR15   CSFGIQMAITACAAILIMSVITSLCWHFDGPWYIRMGWYWCMAKRRQYEKRPENKAYDAF 714
ancaTLR15   CSLGLQMGITAIVAAMFMTVITVLCWRFHGPWYIKMGWYWCMAKRKQYQKSPEDKLYDAF 671
            **:*:**.***  * :.: *:* ***:*.* **::**********:**:* **:* :***

                                        TIR
gagaTLR15   ISYSEHDADWTKEHLLKKLETDGFKICYHERDFKPGHPVLGNIFYCIENSHKVLFVLSPS 770
crpoTLR15   ISYSENDASWTKENLLEKLEIKGFKICYHERDFKPGHPVLGNIFYCIENSHKVLFVLSPS 776
almiTLR15   ISYSENDASWTKENLLEKLETKGFKICYHERDFKPGHPVLGNIFYCIENSHKVLFVLSPS 774
ancaTLR15   VSYSENDAPWTKEILLKNLEANNYRVCYHERDFLPGHPVLGNIFHCIENSHKVLFVLSPS 731
            :****:** **** **::** ..:::******* **********:***************

                                        TIR
gagaTLR15   FVNSCWCQYELYFAEHRVLDENQDSLIMVVLEDLPPDSVPQKFSKLRKLLKRKTYLKWSP 830
crpoTLR15   FVNSCWCQYELYFAEHRVLNENQDSLIMVVLEDLPPNSVPQKFSKLRKLLKRKTYLKWSP 836
almiTLR15   FVNSCWCQYELYFAEHRVLNENQDSLIMVVLEDLPPNSVPQKFSKLRKLLKRKTYLKWSP 834
ancaTLR15   FVNSCWCQYELYFAEHRVLNENQDSLIMIVLEDLPPNSVPQKFSKLRKLLKRKTYLKWSS 791
            ******************:*********:*******:**********************

                  TIR
gagaTLR15   EEHKQKIFWHQLAAVLKTTNEPLV-RAENGPNEDVIEME-- 868
crpoTLR15   EEQKQKIFWHQLTAVLKTSNDPIVLKSKNGLNQDTYEMEFH 877
almiTLR15   EEQKQKIFWHQLTAVLKTSNDPIVLKAENGLNQDTYEMECH 875
ancaTLR15   EEHKQKLFWCQLNAVLKTTNEPMVLDETIELH--------- 823
            **:***:** ** *****:*:*:*       :
```

Suppl. Figure 1. Comparison of TLR15 protein sequences. Amino acid sequences of chicken (gaga), crocodile (cpro), alligator (almi) and anolis (anca) TLR15 were aligned using the Clustal Omega server with default settings. Asterisks (*) indicate identical residues, double square dots (:) indicate highly similar residues, single square dots (.) indicate somewhat similar residues, and bars (–) indicate gaps to complete the sequence alignment. Signal peptide, LRRs, CTLRR, TM and TIR domain are shaded in gray.
