# Supplementary material for: Evolutionary Regression and Species-Specific Codon Usage of TLR15
Source: Front Immunol. 2018 Nov 13;9:2626. doi: 10.3389/fimmu.2018.02626 (PMC6244663; doi:10.3389/fimmu.2018.02626)
Supplement: Supplementary file 2 [file Data_Sheet_2.PDF]

Suppl. Table 1.

Species of which TLR1 subfamily protein sequences were collected from GenBank

Species	Abbreviation	Group	Common name
Danio rerio	dare	actinopterygii (post TS-WGD*)	Zebrafish
Oryzias latipes	orla	actinopterygii (post TS-WGD)	Medaka
Lepisosteus oculatus	leoc	actinopterygii (pre TS-WGD)	Spotted gar
Nanorana parkeri	napa	amphibia	High Himalaya frog
Xenopus tropicalis	xetr	amphibia	Western clawed frog
Callorhinchus milii	cami	chondrichtyes	Australian ghost shark
Homo sapiens	hosa	mammalia	Human
Mus musculus	mumu	mammalia	Mouse
Sus scrofa	susc	mammalia	Pig
Myotis brandtii	mybr	mammalia	Brandt's bat
Orcinus orca	oror	mammalia	Killer whale
Chelonia mydass	chmy	reptilia	Green sea turtle
Chrysemys picta bellii	chpi	reptilia	Painted turtle
Pelodiscus sinensis	pesi	reptilia	Chinese softshell turtle
Alligator mississippiensis	almi	reptilia (archosaur)	American alligator
Alligator sinensis	alsi	reptilia (archosaur)	Chinese alligator
Crocodylus porosus	crpo	reptilia (archosaur)	Salt water crocodile
Gavialis gangeticus	gavga	reptilia (archosaur)	Indian Gharial
Anas platyrhynchos	anpl	reptilia (archosaur, aves)	Mallard duck
Columba livia	coli	reptilia (archosaur, aves)	Rock pigeon
Falco peregrinus	fape	reptilia (archosaur, aves)	Peregrine falcon
Gallus gallus	gaga	reptilia (archosaur, aves)	Chicken
Struthio camelus australis	stca	reptilia (archosaur, aves)	South African ostrich
Taeniopygia guttata	tagu	reptilia (archosaur, aves)	Zebrafinch
Anolis carolinensis	anca	reptilia (lepidosaur)	Green anole lizard
Gekko japonicus	geja	reptilia (lepidosaur)	Schlegel's Japanese gecko
Pogona vitticeps	povi	reptilia (lepidosaur)	Bearded dragon
Protobothrops mucrosquamatus	prmu	reptilia (lepidosaur)	Brown spotted pit viper
Python bivittatus	pybi	reptilia (lepidosaur)	Burmese python
Thamnophis sirtalis	thsi	reptilia (lepidosaur)	Common garter snake
Latimeria chalumnae	lach	sarcopterygii	Coelacanth
Ciona intestinalis	ciin	urochordate (non-vertebrate)	Sea squirt

*TS-WGD: teleost specific whole genome duplication
