# Supplementary material for: Evolutionary Regression and Species-Specific Codon Usage of TLR15
Source: Front Immunol. 2018 Nov 13;9:2626. doi: 10.3389/fimmu.2018.02626 (PMC6244663; doi:10.3389/fimmu.2018.02626)
Supplement: Supplementary file 3 [file Data_Sheet_3.PDF]

Table S2. Identity matrix of full length TLR15 protein sequences

Identity (%)	gagaTLR15	crpoTLR15	almiTLR15	ancaTLR15
gagaTLR15	100	69	70	59
crpoTLR15		100	95	52
almiTLR15			100	61
ancaTLR15				100
