# Supplementary material for: Evolutionary Regression and Species-Specific Codon Usage of TLR15
Source: Front Immunol. 2018 Nov 13;9:2626. doi: 10.3389/fimmu.2018.02626 (PMC6244663; doi:10.3389/fimmu.2018.02626)
Supplement: Supplementary file 4 [file Data_Sheet_4.PDF]

Supplementary Table 3. Percentage (%) and absolute number (Counts) of codons per amino acid in the TLR15 genes of the indicated species in relation to the percentage of codon usage in the human genome. Per amino acid, the most frequent codon in the human genome is color coded in blue and the least frequent codon is color coded in red.

		Percentage in human genome	TLR15							
			G. gallus		*A. carolinensis*		*C. porosus*		*A. mississippiensis*	
Amino acid	codon		%	Counts	%	Counts	%	Counts	%	Counts
ALA	GCA	24,776	45,238	19	25,000	7	41,935	13	39,394	13
	GCG	9,203	0,000	0	3,571	1	0,000	0	0,000	0
	GCC	38,471	23,810	10	39,286	11	12,903	4	21,212	7
	GCT	27,550	30,952	13	32,143	9	45,161	14	39,394	13
ARG	AGA	23,320	48,485	16	39,130	9	48,387	15	42,857	12
	AGG	21,662	27,273	9	17,391	4	25,806	8	32,143	9
	CGA	11,339	0,000	0	13,043	3	3,226	1	7,143	2
	CGG	19,415	9,091	3	13,043	3	9,677	3	7,143	2
	CGC	16,170	6,061	2	4,348	1	3,226	1	3,571	1
	CGT	8,094	9,091	3	13,043	3	9,677	3	7,143	2
ASN	AAC	50,301	49,153	29	56,923	37	31,429	22	32,353	22
	AAT	49,699	50,847	30	43,077	28	68,571	48	67,647	46
ASP	GAC	50,851	46,341	19	53,125	17	48,649	18	42,105	16
	GAT	49,149	53,659	22	46,875	15	51,351	19	57,895	22
CYS	TGC	51,452	55,556	10	42,105	8	25,000	4	35,294	6
	TGT	48,548	44,444	8	57,895	11	75,000	12	64,706	11
GLN	CAA	28,135	41,935	13	55,882	19	51,724	15	50,000	14
	CAG	71,865	58,065	18	44,118	15	48,276	14	50,000	14
GLU	GAA	45,289	63,265	31	67,308	35	73,684	42	76,364	42
	GAG	54,711	36,735	18	32,692	17	26,316	15	23,636	13
GLY	GGA	26,775	32,258	10	23,333	7	41,935	13	41,176	14
	GGG	24,445	25,806	8	30,000	9	19,355	6	17,647	6
	GGC	31,859	25,806	8	30,000	9	19,355	6	23,529	8
	GGT	16,921	16,129	5	16,667	5	19,355	6	17,647	6
HIS	CAC	55,719	52,174	12	48,387	15	38,462	10	33,333	10
	CAT	44,281	47,826	11	51,613	16	61,538	16	66,667	20
ILE	ATA	18,421	27,273	15	22,000	11	35,938	23	38,710	24
	ATC	43,837	29,091	16	36,000	18	21,875	14	17,742	11
	ATT	37,742	43,636	24	42,000	21	42,188	27	43,548	27
LEU	CTA	7,531	10,924	13	5,217	6	12,613	14	10,811	12
	CTG	37,520	34,454	41	26,957	31	17,117	19	21,622	24
	CTC	18,439	16,807	20	16,522	19	17,117	19	16,216	18
	CTT	14,224	15,966	19	16,522	19	16,216	18	16,216	18
	TTA	8,724	10,924	13	13,043	15	25,225	28	19,820	22
	TTG	13,561	10,924	13	21,739	25	11,712	13	15,315	17
LYS	AAA	45,960	60,417	29	60,714	34	75,439	43	77,586	45
	AAG	54,040	39,583	19	39,286	22	24,561	14	22,414	13
MET	ATG	100,000	100,000	15	100,000	25	100,000	14	100,000	12
PHE	TTC	51,173	45,652	21	48,571	17	26,531	13	25,000	12
	TTT	48,827	54,348	25	51,429	18	73,469	36	75,000	36
PRO	CCA	29,335	37,500	21	48,649	18	42,857	18	46,341	19
	CCG	10,118	8,929	5	0,000	0	2,381	1	0,000	0
	CCC	30,528	23,214	13	24,324	9	26,190	11	24,390	10
	CCT	30,019	30,357	17	27,027	10	28,571	12	29,268	12
SER	AGC	23,091	28,571	24	24,359	19	25,610	21	21,687	18
	AGT	16,029	14,286	12	19,231	15	17,073	14	19,277	16
	TCA	16,228	15,476	13	19,231	15	24,390	20	22,892	19
	TCG	4,887	2,381	2	7,692	6	0,000	0	2,410	2
	TCC	20,391	17,857	15	11,538	9	8,537	7	9,639	8
	TCT	19,373	21,429	18	17,949	14	24,390	20	24,096	20
THR	ACA	30,155	36,585	15	42,105	16	44,186	19	45,238	19
	ACG	10,569	9,756	4	0,000	0	9,302	4	9,524	4
	ACC	33,186	24,390	10	21,053	8	18,605	8	14,286	6
	ACT	26,090	29,268	12	36,842	14	27,907	12	30,952	13
TRP	TGG	100,000	100,000	10	100,000	11	100,000	10	100,000	11
TYR	TAC	53,245	52,174	12	52,381	11	40,625	13	44,828	13
	TAT	46,755	47,826	11	47,619	10	59,375	19	55,172	16
VAL	GTA	12,803	29,545	13	23,256	10	35,556	16	31,915	15
	GTG	44,514	29,545	13	32,558	14	24,444	11	25,532	12
	GTC	23,046	15,909	7	18,605	8	6,667	3	6,383	3

	GTT	19,637	25,000	11	25,581	11	33,333	15	36,170	17
STOP	TAA	27,439	0,000	0	100,000	1	0,000	0	0,000	0
	TAG	21,951	0,000	0	0,000	0	100,000	1	100,000	1
	TGA	50,610	100,000	1	0,000	0	0,000	0	0,000	0
Total				869		824		878		876
Sum of most frequent codons			35,67	310	34,83	287	26,42	232	25,91	227
Sum of least frequent codons			27,62	240	27,67	228	36,22	318	36,30	318
