# Supplementary material for: Evolutionary Regression and Species-Specific Codon Usage of TLR15
Source: Front Immunol. 2018 Nov 13;9:2626. doi: 10.3389/fimmu.2018.02626 (PMC6244663; doi:10.3389/fimmu.2018.02626)
Supplement: Supplementary file 5 [file Data_Sheet_5.PDF]

Suppl. Table 3

Primers used for cloning reptile TLR15

Product	Primer	Sequence (5'- 3')
ancaTLR15	Forward	ATGGGAACTTTCATCCACAGTCTGCA
	Reverse	ATGCAGCTCGATTGTTTCATCC
KpnI-kozak-ancaTLR15-3xHA-overlap	Forward	CCGGTACCGCCACCATGGGAACTTTCATCCACAGTCTGCA
	Reverse	CATATGGGTAGCGGCCGCTATGCAGCTC
ancaTLR15-overlap-3xHA-PacI	Forward	GCATAGCGGCCGCTACCCATATGACGTTCCAG
	Reverse	CCTTAATTAATCAAGCGTAGTCAGGTACATCG
KpnI-kozak-crpoTLR15-NotI	Forward	CCGGTACCGCCACCATGGGTATCCTCATCAGAGTTC
	Reverse	CCGCGGCCGCGGTGAAATTCCATCTCATATGTATCTTG
KpnI-kozak-almiTLR15-NotI	Forward	CCGGTACCGCCACCATGGGTATCCTCATTCTTCG
	Reverse	CCGCGGCCGCGGTGGCATTCCATCTCATATG
